# The dilemma of bacterial expansins evolution. The unusual case of *Streptomyces acidiscabies* and *Kutzneria* sp. 744

**DOI:** 10.1080/19420889.2018.1539612

**Published:** 2018-11-04

**Authors:** Vinicio Armijos-Jaramillo, Daniela Santander-Gordón, Eduardo Tejera, Yunierkis Perez-Castillo

**Affiliations:** a Grupo de Bio-Quimioinformática, Universidad de Las Américas, Quito, Ecuador; b Carrera de Ingeniería en Biotecnología, Facultad de Ingeniería y Ciencias Aplicadas, Universidad de Las Américas, Quito, Ecuador; c Facultad de Ciencias Naturales y Ambientales, Universidad Internacional SEK, Quito, Ecuador; d Ciencias Físicas y Matemáticas-Facultad de Formación General, Universidad de Las Américas, Quito, Ecuador

**Keywords:** Expansins, *Streptomyces acidiscabies*, *Kutzneria* sp. 744, horizontal gene transfer, evolution

## Abstract

Expansins are a superfamily of proteins mainly present in plants that are also found in bacteria, fungi and amoebozoa. Expansin proteins bind the plant cells wall and relax the cellulose microfibrils without any enzymatic action. The evolution of this kind of proteins exposes a complex pattern of horizontal gene transferences that makes difficult to determine the precise origin of non-plant expansins. We performed a genome-wide search of inter-domain horizontal gene transfer events using *Streptomyces* species and found a plant-like expansin in the *Streptomyces acidiscabies* proteome. This finding leads us to study in deep the origin and the characteristics of this peculiar protein, also present in the species *Kutzneria* sp.744. Using phylogenetic analyses, we determine that indeed *S. acidiscabies* and *Kutzneria* sp.744 expansins are located inside the plants expansins A clade. Using secondary and tertiary structural information, we observed that the electrostatic potentials and the folding of expansins are similar, independently of the proteins’ origin. Using all this information, we conclude that *S. acidiscabies* and *Kutzneria* sp.744 expansins have a plant origin but differ from plant and bacterial canonical expansins. This finding suggests that the experimental research around this kind of expansins can be promissory in the future.

## Introduction

*Streptomyces* species are filamentous prokaryotes characterized by their production of multinucleated mycelium that colonizes and penetrates organic matter in the soil [1]. More than 600 species have been identified as members of this genus [2] that is also known to produce secondary metabolites like antibiotics, antifungals, anticancer agents and virulence factors [3].

*Streptomyces acidiscabies* was first described as a bacteria causing acid scab symptoms indistinguishable from the ones caused by *Streptomyces scabies* [4,5]. *S. acidiscabies* was detected as the potato scab causing agent in USA [6] and China [7]. Also, it has been reported that this acid scab pathogen has another hosts like carrot, beet and radish [8]. The main characteristic of this species is its ability to grow at pH = 4 in culture and at pH = 4.5 in soil [6]. *S. acidiscabies* and the group of bacteria that causes the potato common scab (e.g. *S. europaeiscabiei, S. stelliscabiei, S. scabies, S. turgidiscabies*) have generated important economic losses around the world [9,10].

*Kutzneria* species are part of a narrow genus of the *Pseudonocardiaceae* family, even though they were placed in the *Streptosporangiacea* genus in the first place. Only eight species have been described in this genus and secondary metabolism gene clusters have been identified in some of them [11]. Particularly, *Kutzneria* sp. 744 demonstrate to produce several metabolites that have antagonistic effects on the growth of root pathogens like *Pythium undulatum, Ceratobasidium bicorne* and *Fusarium avenaceum* [12]. *Kutzneria* sp. 744 was isolated from the mycorrhizal root tips of Norway spruce seedlings (information available in the BioProject PRJNA38,053 of NCBI: https://www.ncbi.nlm.nih.gov/bioproject/38053)

Expansins are small cell wall proteins composed of 225 to 300 amino acid residues and known to loosen plant cell walls in a pH dependent and non-enzymatic manner [13]. These proteins are made-up of an initial signal peptide and two domains (named 1 and 2) [14]. Even when they were first identified in plants [15,16], they have been found in other organisms like bacteria, fungi and amoeba. In plants, they play several roles in morphogenetic processes like germination, fruit ripening, growth of pollen tube and root hairs, defoliation and others that have not yet been discovered [17]. Also, expansins are catalysts of cell wall enlargement, an important function that has been well documented by several authors [16,18–20]. Based on phylogenetic analyses, plants expansins are classified into four families: α-expansin or expansin A (EXPA), β-expansin or expansin B (EXPB), α-like expansin or expansin-like A (EXLA) and β – like expasin or expansin-like B (EXLB) [21].

Expansin-like proteins have been found in bacteria, mainly in Proteobacteria and Actinobacteria phyla, clustering into four distinct expansin-like X subgroups [22]. As in plants, bacterial expansins present a wall-loosening action with the difference that their activity is weaker, a property thought to be important in pathogens that wants to avoid triggering plant defenses [23]. Also, it has been demonstrated that they function as a cellulase activity enhancers, a feature that can be exploited in the lignocellulosic biomass degradation field [22]. A resemblance between plant expansins and bacterial expansins endoglucanases and cellulose binding domains has been identified [14,24–28]. This similarity was confirmed by the crystallographic structure of a maize pollen β-expansin that contains the two-domain structure present in all expansins previously described [29]. These structural similarities and the scarce phylogenetic distribution of expansins outside Viridiplantae kingdom leads to propose their origin in plants followed by several and independent horizontal gene transfer events to bacteria, fungi and Protista [30].

In this work we performed and inter-domain horizontal gene transfer search using the proteome of *Streptomyces acidiscabies* NCPPB 445 as query. Then, we found an expansin that resembles plant expansins A in this species and in *Kutzneria* sp. 744 (only in these two organisms). We collect phylogenetic and structural evidence to elucidate the complex evolutionary history of this kind of bacterial proteins.

## Results and discussion

We performed an inter-domain horizontal gene transfer search, using *Streptomyces* proteins as query. This resulted in the finding of an expansin protein in *S. acidiscabies* NCPPB 445 (also detected in *Kutzneria* sp. 744) that resemble plant expansins. These bacterial proteins are more similar to plant expansins A (EXPA) (43 % of similarity and 100 % of coverage with the best plant BLAST hit), than to bacterial expansin-like (32,5% of similarity and 85% of coverage with the best bacterial BLAST hit) also called EXLX following the nomenclature of Kende et al. (2004) [21]. The presence of these plant expansin-like proteins was also previously observed by Georgelis et al., 2015 and Nikolaidis, Doran, & Cosgrove, 2014 [30,31]. With the information available in the public databases (may 2018), we identified this kind of proteins only in *S. acidiscabies* strains and *Kutzneria* sp. 744. Our BLAST search was not able to find close related homologues in other bacteria, including *Streptomyces* and *Kutzneria* genera. In order to differentiate the *S. acidiscabies* and *Kutzneria* expansins from the rest of bacterial, fungal and plant variants of this superfamily, we decide to call them Bacterial plant-like expansins A (BPLEA). The presence of these BPLEAs was observed in 9 strains of *S.acidiscabies* but only in the strain 744 of *Kutzneria* sp. All the copies in *S. acidiscabies* are identical at amino acid and DNA level except for the copy of the strain NCPPB 4445 (WP_050370046). The expansin version in this strain has only 53,3 % identity with their putative orthologues in the others strains.

Additionally, we study the potential presence of BPLEAs genes inside a genomic island. To perform this task, we used genomic contigs of *S. acidiscabies* NCPPB 4445, *S. acidiscabies* a10 and *Kutzneria* sp. 744 to detect putative alien regions by surrogate methods (comparison of features lengthwise the genome). The program Alien_Hunter [6] detected alien regions that includes the *BPLEA* genes for *S. acidiscabies* NCPPB 4445 and *Kutzneria* sp. 744 but not for *S. acidiscabies* a10. The program IslandViewer4 [7] predicted a BPLEA gene inside a genomic island located only in *Kutzneria* sp. 744 but not within the two strains of *S. acidiscabies* (Table 1). However, the alien predicted regions include genes without evidence of inter-domain HGT (inferred by phyletic methods, data not shown). The genomic islands are associated with the mobility of chromosomal DNA [8], and the localization of *BPLEA* genes inside of these portions of the genomes is highly congruent. However, we take these results with caution because surrogate methods have been demonstrated to be highly unspecific and insensible to detect HGT cases [4]. This is because intragenomic variation can be so broad that it can confound indigenous regions with aliens’ ones. Intragenomic variation can be produced by stochastic events or by highly expressive genes with codon bias [4,5].

**Table 1.** *BPLEA* genes predicted inside genomic islands.

Species	Genomic contig^a^	*BPLEA* gene^a^	Relative gene position to the contig	Genomic Island predicted region-Alien_Hunter^+^	Genomic Island predicted region-IslandViewer4^+^
*S. acidiscabies* NCPPB 4445	NZ_KQ257808	IQ63_RS07865	47,596–48,366	45,000–50,000	21,391–37,030
*S. acidiscabies* a10	BCMK01000043	a10_05000	18,945–19,709	22,500–27,500	74,467–77,970
*Kutzneria sp. 744*	NZ_KK037166	KUTG_RS02170	488,980–489,726	487,500–492,500	465,288–531,136

^a^NCBI’s locus Id

^+^Predicted region that includes *BPLEA* gene or the nearest predicted region to the gene

## Isoelectric point and secondary structure analysis of BEAPLs

To in-deep explore the similarity found in the primary structure of the proteins, we predicted their domains and motifs using Interproscan [32]. We found in BPLEA proteins the InterPro term IPR002963, whose phylogenetic distribution shows that it is abundant in plant proteins. This term is also present in the amoeba *Acanthamoeba castellanii* str. Neff while in prokaryotes it has been only detected in *Streptomyces acidiscabies* and *Kutzneria* sp. 744. The phylogenetic distribution of the term IPR002963 supports the resemblance of BPLEA proteins to plants expansins. This term was not detected in any other bacterial EXLX protein recovered from our BLAST search. BPLEA proteins were classified into the PTHR31867 family of Panther classification system [33], the same as plant expansin A proteins. The sequences in bacteria most similar to BPLEAs were classified as PTHR31836 Panther family, showing once again the proximity of BPLEAs to plant expansins. All the expansins (BPLEA, EXLX and EXPA) were predicted to have a signal peptide and to contain the EXPANSIN_EG45 (Expansin/allergen_DPBB/glycosyl hydrolase 45- IPR007112) and EXPANSIN_CBD (cellulose-binding-like domain- IPR007117) domains. This is the same structure observed in canonical families of plant expansins [14]. However, the isoelectric point of these motifs varies in the proteins included in our comparison (Table 2). These differences could be important to determine the mechanism of action and the specificity to the substrates that the expansins bind. A good example of the effect of the pH in the mechanism of expansins was observed in BsEXLX1 (YOAJ_BACSU) of *Bacillus subtilis* that is able to bind either cellulose or pectin. Other example is PcExl1 (W5VT34_PECCA) of *Pectobacterium carotovorum* that is able to bind cellulose only [34]. The PcExl1 protein functions at lower pH than the BsEXLX1 protein. This observation is in agreement with our calculation that the isoelectric points of these two molecules are considerable different (Table 2). In fact, the isoelectric points of the PcExl1 domains are lower than in plant expansins. Especially, the isoelectric point of the CBD domain of WP_050370046 (*S. acidiscabies* NCPPB 445) is particularly low (6.8), comparable with the value for the CBD of PcExl1 of *Pectobacterium carotovorum* (7.67). These values are distant from the isoelectric points of EXPAs or others EXLXs. Given that the CBD domain is in direct contact with cellohexaose, this differences could determine different mechanisms of action or substrates specificities among expansins [35].

**Table 2.** Isoelectric point of expansin domains from proteins compared in this study.

			Expansin domains	
Expansin family	NCBI/Uniprot ID	Species	DPBB domain (isoelectric point)	CBD domain (isoelectric point)
BPLEA	WP_050370046	*Streptomyces acidiscabies* NCPPB 4445	4.78	6.8
	GAQ55178	*Streptomyces acidiscabies* a10	5	11.48
	WP_043714506	*Kutzneria sp. 744*	4.55	11.61
EXPA	NP_195846*	*Arabidopsis thaliana*	7.66	11.01
EXLX	WP_074473674*	*Micromonospora carbonacea*	7.95	10.04
PcExl1	W5VT34_PECCA	*Pectobacterium carotovorum*	4.06	7.67
BsEXLX1	YOAJ_BACSU	*Bacillus subtilis*	5.06	10.34

*Sequence with high sequence similarity to BPLEA in plant and bacteria

## Phylogenetic analysis and evolutionary scenery

**Figure 1.**
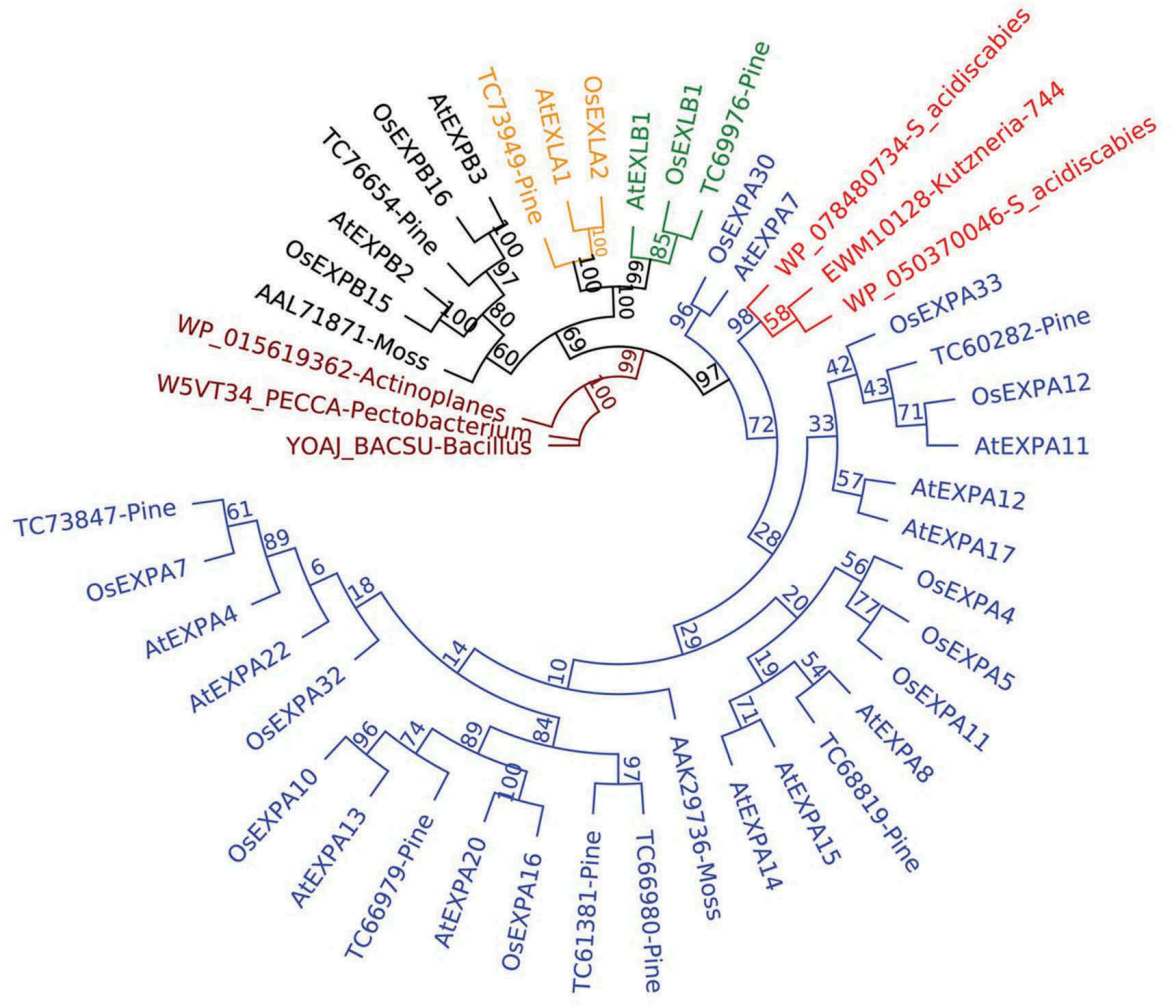
Maximum likelihood tree of plant and bacteria expansins. Blue labels correspond to plant expansins A, light red labels correspond to BPLEAs, black labels correspond to plant expansins B, orange labels correspond to plant expansins-like A, green labels correspond to plant expansins-like B and red labels correspond to bacterial (EXLX) expansins. Non-parametric bootstrap percentages are shown on the internal nodes.

The expansin superfamily have a scatter distribution in the tree of life, this is, it is broadly present in plants, scarce in bacteria and fungi, and present only in a few amoebozoa species [30]. This scenario is compatible with a complex horizontal gene transfer (HGT) followed by differential gene losses. In bacteria and fungi the presence of expansins is strongly correlated to plant-associated species [30], thus, the differential gene loss panorama is congruent with this observation. Despite the similarities among bacterial and plant expansins, we found that BPLEAs are more similar to plant sequences than to any other expansin (including bacteria). To confirm this result we reconstructed the BPLEA tree using the most similar sequence in bacteria and the canonical subfamilies of plant expansins reported in Sampedro & Cosgrove (2005)^14^ Figure 1.Consistently with the BLAST analysis, BPLEAS are more similar to plant expansins than to bacterial ones. To explain this observation, it is necessary to extend the HGT idea proposed in Nikolaidis et al. (2014) [30] about the origin of expansins in bacteria. According to these authors, the expansins appeared once in the evolution and then these proteins were trespassed to distant organisms by HGT. However, we need a more recent HGT event to explain the larger similarity of BPLEAs to plant expansins in comparison to bacterial ones. This idea was proposed in Georgelis et al. (2015) [31] but the complexity of the phylogenetic pattern deserves a deep explanation.

Following the HGT hypothesis, the low conservation between BPLEAs and EXPA suggests an ancient horizontal transference that precedes at least the divergence between flowering plants and Pinidae (estimated time 313 million years ago, MYA). That implies the losing of BPLEAs in most part of the modern species of *Streptomyces* and/or *Kutzneria* lineages, including bacterial species associated with plants and with very similar lifestyle to *S. acidiscabies* (e.g *S. scabies* or *S. turgiscabies*). This is unlikely because species with a similar lifestyle could be benefit from a BPLEA protein as much as *S. acidiscabies*. Another option is the transference of BPLEA approximately in the last common ancestor between *Streptomyces* and *Kutzneria*. These genera shared a common ancestor around 1278.1 MYA (range 1176.2–1380.0 MYA) (see methods). This hypothesis is unlikely, given that at least 450 modern species of Streptomycetales and Pseudonocardiales with a complete genome available in the NCBI (https://www.ncbi.nlm.nih.gov/genome/) would lost this gene over their evolution. The third option is a very recent HGT from plants (near to the apparition of modern *S. acidiscabies* and *Kutzneria* sp. 744), but the lack of sequence conservation (around 50 % of identity) and the phylogenetic distribution make this hypothesis difficult to support.

Regarding the number of Expansins A duplication in their protein tree, it is highly plausible the loss of several copies in the current species. We can also see in Figure 1 the divergence between some copies of Expansin A, for example, between OsEXPA30 and AtEXPA7 of *Oryza sativa* and *Arabidopsis thaliana* respectively. This reflects a differential pressure under the members of expansins superfamily in plants. In this scenario, the BPLEAS could originate in one of the loosed copies of plant expansins A or in a highly divergent copy of the family, followed by a rapid evolution in bacteria too. Both hypotheses explain the low similarity observed between BPLEAS and plant expansins A, which in turn explains their low similarity with bacterial expansins (EXLX). With the information available at this moment in the databases it is not possible to distinguish among these two alternatives. We expect to find a solution when more plant and bacterial proteomes become available.

About the BPLEALs evolution in bacteria, the primary transference from plants should occur to one species (*S. acidiscabies* or *Kutzneria* sp. 744) and then laterally transferred to the other. This scenario is necessary to explain the relatively low similarity (69%) between BEAPL of *S. acidiscabies* and *Kutzneria* sp. 744. We expect to find in the future other members of BEAPLs in bacterial species with a similar evolutionary history.

## Structural analysis

As has been pointed out in previous research, expansins are composed by two domains [34]. The N-terminal domain is structurally related to family-45 glycosyl hydrolase (GH45), but without the later’s β 1,4 glucanase activity. On the other hand, the C-terminal domain of expansins has been demonstrated to be responsible for the binding to cellulose in both plants and bacteria and it is known as the Cellulose Binding Domain (CBD) [35]. In addition, both domains need to act together for cell wall loosening [36].

The 3D structure of an expansin from *Bacillus subtilis* in complex with cellulose has been solved [35]. This study showed that expansins CBD bind cellulose mainly through hydrophobic interactions between receptor’s aromatic residues and the pyranose rings of cellulose, specifically through CH-π interactions. In addition, some hydrogen bonds between the CBD domain of expansin and cellulose’s hydroxyl groups were observed. Mutational studies also confirmed that the presence of aromatic residues on the cellulose recognition site of the expansins CBDs is essential for cellulose binding to expansins.

To study the 3D structure of the BPLEAs, we analyze in-deep the sequences WP_050370046 and GAQ55178 of *S. acidiscabies*, WP_043714506 of *Kutzneria* sp. 744 and one of the most similar sequences to BPLEA in plants XP_010938009 of *Elaeis guineensis*. The PSIPRED server predicted all query sequences as belonging to the expansins superfamily. In all cases either the EXPB1, a beta-expansin and group-1 pollen allergen from maize (PDB code 2hcz) or Phl p 1, a Major Timothy Grass Pollen Allergen (PDB code 1n10) were selected as the best templates for modeling them with a p-value lower than 10^−4^. Given the importance of the CBD of expansins for cell wall recognition, modeling studies focus on this domain.

In contrast to whole sequences, the PSIPRED server predicted the WP_050370046 C-terminal domain as an expansin CBD with medium confidence (p = 0.003). The GAQ55178, WP_043714506 and XP_010938009 C-terminal domains were all predicted as expansin CBDs with high or certain levels of confidence (p < 10^−3^). In all cases, the CBD domain of the Phl p 1 Grass Pollen Allergen (PDB code 1n10) was identified as the best hit structure. The alignments between the query proteins and PDB 1n10 produced by PSIPRED were used for homology modeling.

**Figure 2.**
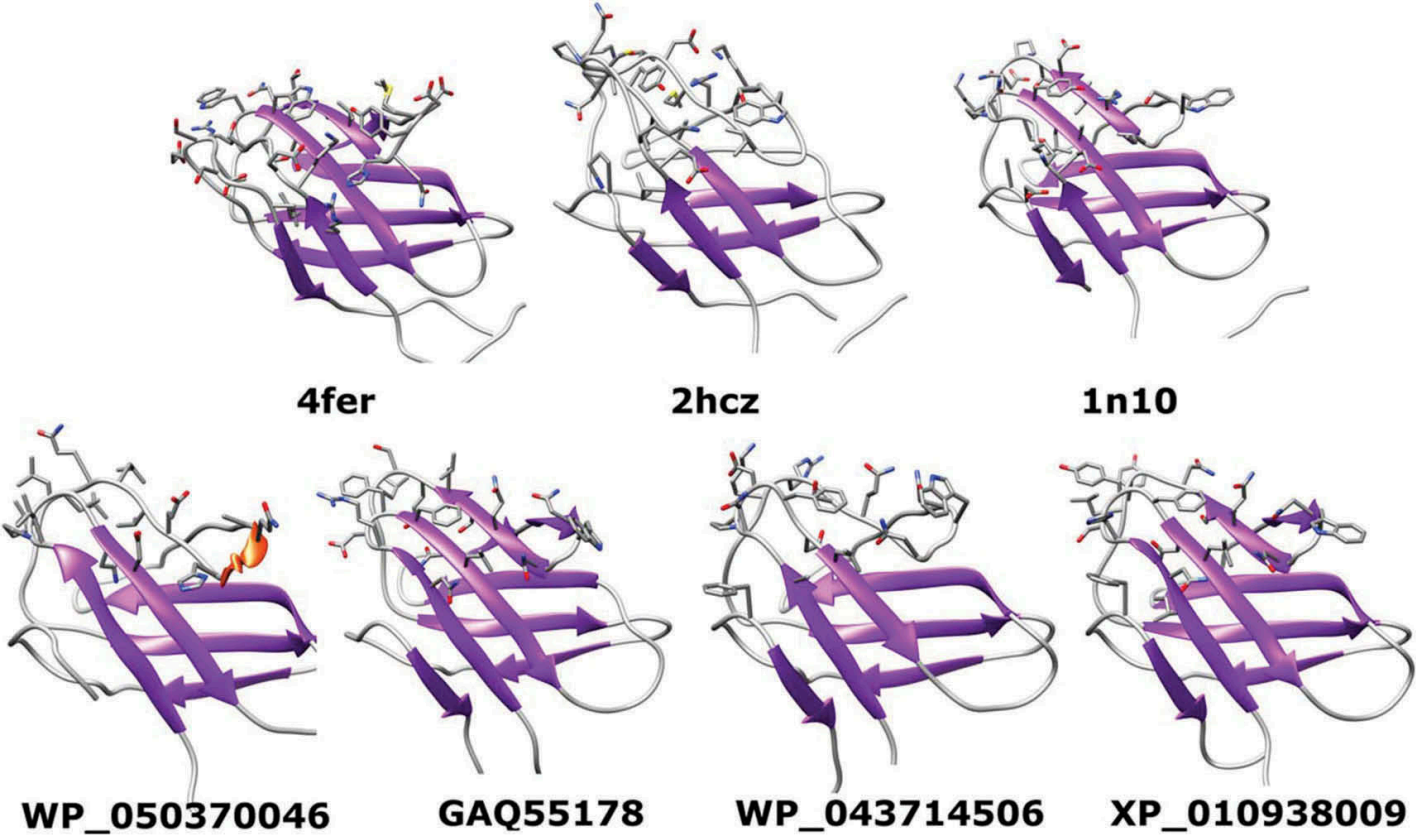
Comparison between the known structures of expansins CDBs (top) and the homology models of the query sequences (bottom).

Overall, obtained homology models resemble the general fold of expansins. However, a comparison between the cellulose binding sites of the CBD of expansins with known structures and the query sequences (Figure 2) reveals some interesting facts. The expansins CBDs of the PDB structures 4fer (*Bacillus subtilis* EXLX1), 1n10 and 2hcz were selected for this comparison. From Figure 2 it can be seen that both expansins with known structures and query proteins contain LYS, GLN, ASN, ARG, GLU and ASP residues. These residues can make direct and water-mediated hydrogen bonds with the hydroxyl groups of cellulose. However, these potential hydrogen bonds are not essential for cellulose-binding [35].

As previously discussed, the presence of aromatic residues at the cellulose-binding site of the expansins CBD is critical for cellulose binding. From Figure 2, it can be seen that this condition is met for the experimentally solved expansins structures as well as for models of GAQ55178, WP_043714506 and XP_010938009. However, no aromatic residue is present at the cellulose-binding site of WP_050370046 CBD.

**Figure 3.**
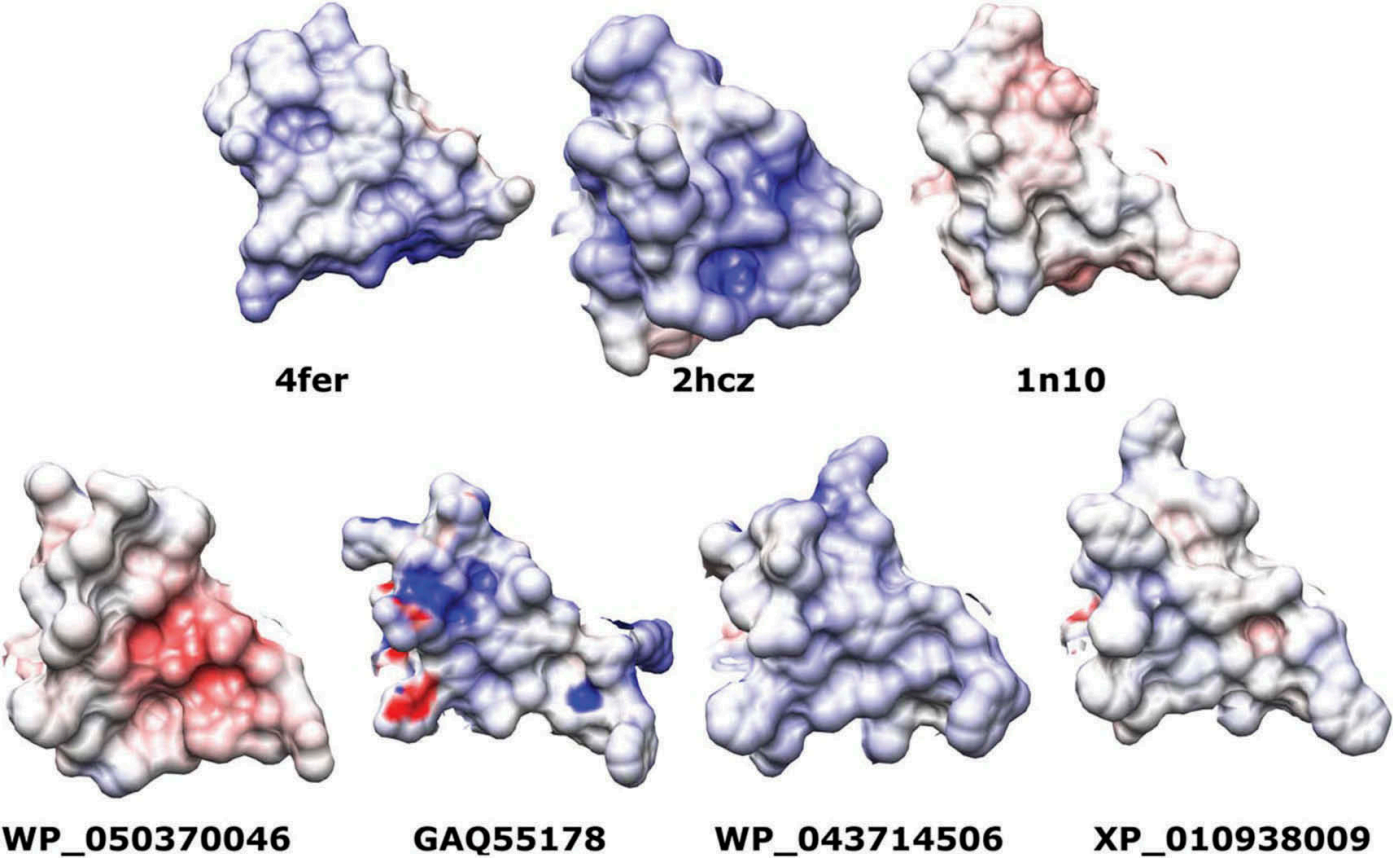
Comparison between the electrostatic potentials of expansins CBDs with known structures (top) and the homology models of the query sequences (bottom). The gradient of color varies from blue (electropositive) to red (electronegative) passing by the neutrality represented in white.

To get further insights into the possible binding of cellulose to the CBD of the query proteins, we examined their electrostatic potential at the cellulose-binding site. Electrostatic potentials for the query proteins as well as for the expansins with known 3D structures are shown in Figure 3. The comparison of the electrostatic potentials of these proteins shows that for known expansins structures, the electrostatic potential at the cellulose-binding site is predominately neutral or slightly positive. This rule also holds for query proteins GAQ55178, WP_043714506 and XP_010938009 and agrees with the hydrophobic nature of cellulose. The observed electropositive potentials can be related to the presence of residues potentially acting as hydrogen bond donors to the hydroxyl groups of cellulose. The presented data indicate that GAQ55178, WP_043714506 and XP_010938009 can be considered as functional members of the expansin superfamily.

In the case of WP_050370046, the electrostatic potential at the cellulose-binding site is predominately electronegative. This observation, together with the low confidence provided by the PSIPRED server during fold recognition, the unusual low isoelectric point (Table 2) and the lack of aromatic residues at the cellulose-binding site are striking. These facts could indicate a miss-annotation of WP_050370046 on the sequences databases. An alternative hypothesis is that WP_050370046 belongs to other type of proteins also sharing a GH45 domain. Another possibility is that WP_050370046 is involved into a pseudogenization process.

Subtle electrostatic potential differences were observed between plant, bacterial (EXLX) expansins and BPLEAs. These changes could have an effect in cellulose binding affinity, but we should consider that all the models were predicted using EXPLB structures. The modeling can be improved when EXPLA structures become available in the Protein Data Bank.

## Conclusion

The expansins that we called BPLEAs have a complex evolutionary history. The lack of close related homologues outside *S. acidiscabies* and *Kutzneria* sp. 744 is intriguing. BEAPLs are clearly more similar to plant expansins than bacterial ones, despite the structural similarities between all the members of the expansin superfamily explored in this study. In that sense, we propose that BEAPLs were originated in a recent HGT event (more recent than the origin of non-plant expansins) from a missing copy of modern plant expansins A (EXPLA). Posterior the HGT, each copy of BPLEA have been rapidly adapted to each species (this explains the differences observed within BPLEAs). Altogether, this panorama explains the sequence similarity and the structural characteristics in-between plant and bacterial expansins observed in BEAPLs. We expect to have access to more plants and bacterial genomes in the future to validate, ameliorate or reformulate the hypotheses proposed in this work. However, the uniqueness of BEAPLs turns these proteins interesting for further experimental research and applications in biotechnology.

## Methods

### Inter-domain HGT search

Each protein predicted from *Streptomyces acidiscabies* NCPPB 445 genome (bioproject: PRJN255692) served as a query to perform a BLASTp search against the UniProtKB (TrEMBL+ Swiss-Prot) database. All the accessions available in UniProtKB (dec-2017) were uploaded into a MySQL database and the taxonomy of each Blast hit was retrieved from this database using a custom script. Then, we selected the queries that showed at least 80% of Blast hits with a different taxonomic status rather than bacteria. The threshold was determined according to Armijos-Jaramillo, Santander-Gordón, Soria, Pazmiño-Betancourth, & Echeverría (2016) [37]. With the selected *S. acidiscabies* proteins we reconstructed phylogenetic trees using their best BLAST hits. The multiple sequence alignment was performed with MAFFT and the tree was reconstructed with PhyML, using LG amino-acid replacement matrix, SH branch support and the default values for the rest of parameters. To select candidates with a proper HGT pattern, we manually evaluated all tree topologies generated.

Additionally, we used the programs Alien_Hunter [38] and IslandViewer4 [39] to detect the presence of genomic islands associated to HGT genes. The genomic assembly contigs of *S. acidiscabies* NCPPB 4445 (NZ_KQ257808), *S. acidiscabies* a10 (BCMK01000043) and *Kutzneria* sp. 744 (whole genome assembly, NZ_KK037166) were used.

### Phylogenetic reconstruction of bacterial plant-like expansins a (BPLEA)

The expansins’ phylogenetic tree of *S. acidiscabies* and *Kutzneria* sp. 744 was reconstructed using the plant expasins established by Sampedro & Cosgrove (2005) [14]. Additionally, we use the sequences BsEXLX1 (YOAJ_BACSU) of *Bacillus subtilis*, PcExl1 (W5VT34_PECCA) of *Pectobacterium carotovorum* (both with crystal structures available in the Protein Data Bank) and WP_015619362 of *Actinoplanes* sp. N902-109 (one of the most similar bacterial sequence to the query) as members of bacterial expansins. A multiple sequence alignment was performed in MAFFT [40] (using default parameters) and the tree was reconstructed with PhyML [41], using LG amino-acid replacement matrix, SH branch support, with the proportion of invariable sites and Gamma distribution parameter estimated by the program. To evaluate the impact of the alignment in the tree topology, several methods of multiple sequence alignment and alignment edition were performed. Manual edition, trimAL v1.2 (default parameters) [42] and Gblocks 0.91b (default parameters) [43] were used to evaluate the effect of alignment edition in the tree. In addition to MAFFT, we used ClustalW 2 (default parameters) [44] and MUSCLE 3.8 (default parameters, eight iterations) [45] to calculate multiple sequence alignments before the tree reconstruction.

To ensure the presence of expansin plant-like proteins in other strains of *S. acidiscabies* and *Kutzneria*, we use the Pathosystems Resource Integration Center (PATRIC) database [46]. This database contains the complete genome information of strains 84–104, 85–06, 98–48, 98–49, FL01, NCPPB 4445, NRRL B-16,524, a10 of *S. scabies* and the species *Kutzneria* sp. 744 and *Kutzneria albida* (strains DSM 43,870 and NRRL B-24,060)

To calculate the divergence time between ﻿Streptomycetales (*S. acidiscabies* order) and ﻿Pseudonocardiales (*Kutzneria* order) and between flowering plants and Pinidae we used the estimation of TimeTree [47].

### 3-D modeling and analysis

Sequences were submitted to the PSIPRED server for secondary structure prediction and fold recognition with the pGenTHREADER algorithm [48–50]. This approach uses profile-profile alignments and the predicted secondary structure of the query sequence to produce accurate alignments between it and proteins with known structures. The PSIPRED server also provides a confidence of the predictions made.

Molecular visualization, figures and calculations were performed using UCSF Chimera [51]. Homology models were developed using the MODELLER software [52] executed from Chimera’s MODELLER interface. The best models were selected according to the DOPE and GA341 scores. Electrostatic potentials were computed with the APBS program [53] using Chimera’s plugin. The three-dimensional structures of expansins were obtained from the Protein Data Bank [54].

## References

[1] Chater KF. *Streptomyces* inside-out: a new perspective on the bacteria that provide us with antibiotics. Philos Trans R Soc B Biol Sci. 2006;361:761–768. 10.1098/rstb.2005.1758 PMC1609407 16627293

[2] Labeda DP Multilocus sequence analysis of phytopathogenic species of the genus *Streptomyces*. Int J Syst Evol Microbiol. 2011;61:2525–2531. 21112986 10.1099/ijs.0.028514-0

[3] Hwang K-S, Kim HU, Charusanti P, et al Systems biology and biotechnology of *Streptomyces* species for the production of secondary metabolites. Biotechnol Adv. 2014;32:255–268. 24189093 10.1016/j.biotechadv.2013.10.008

[4] Lambert DH, Loria R *Streptomyces acidiscabies* sp nov. Int J Syst Evol Microbiol. 1989;39:393–396.

[5] Manzer FE, McIntyre GA, Merriam DC TB85: a new potato scab problem in maine. Life Sci Agric Exp Stn Tech Bull [Internet]. 1977;85 Available from: https://digitalcommons.library.umaine.edu/aes_techbulletin/114

[6] Loria R, Bukhalid RA, Fry BA, et al Plant pathogenicity in the genus *Streptomyces*. Plant Dis. 1997;81:836–846. 10.1094/PDIS.1997.81.8.836 30866367

[7] Zhao WQ, Yu XM, Liu DQ First report of *Streptomyces acidiscabies* causing potato scab in China. Plant Pathol. 2010;59:405.

[8] Lambert DH First report of additional hosts for the acid scab pathogen *Streptomyces acidiscabies*. Plant Dis. 1991;75:750.

[9] Gong C, Yang MP, Yu DC, et al First report of *Streptomyces caviscabies* causing common scab on potato in China. Plant Dis. 2017;101:1316.

[10] Loria R, Kers J, Joshi M Evolution of plant pathogenicity in *Streptomyces*. Annu Rev Phytopathol. 2006;44:469–487. 16719719 10.1146/annurev.phyto.44.032905.091147

[11] Rebets Y, Tokovenko B, Lushchyk I, et al Complete genome sequence of producer of the glycopeptide antibiotic Aculeximycin *Kutzneria albida* DSM 43870T, a representative of minor genus of *Pseudonocardiaceae*. BMC Genomics. 2014;15:1. 25301375 10.1186/1471-2164-15-885 PMC4210621

[12] Broberg A, Menkis A, Vasiliauskas R Kutznerides 1-4, depsipeptides from the actinomycete *Kutzneria* sp. 744 inhabiting mycorrhizal roots of *Picea abies* seedlings. J Nat Prod. 2006;69:97–102. 16441076 10.1021/np050378g

[13] Fukuda H Plant cell wall patterning and cell shape. Hoboken (NJ): John Wiley & Sons; 2014.

[14] Sampedro J, Cosgrove DJ The expansin superfamily. Genome Biol. 2005;6:242. 16356276 10.1186/gb-2005-6-12-242 PMC1414085

[15] Li Z-C, Durachko DM, Cosgrove DJ An oat coleoptile wall protein that induces wall extension in vitro and that is antigenically related to a similar protein from cucumber hypocotyls. Planta. 1993;191:349–356.

[16] McQueen-Mason S, Durachko DM, Cosgrove DJ Two endogenous proteins that induce cell wall extension in plants. Plant Cell. 1992;4:1425–1433. 11538167 10.1105/tpc.4.11.1425 PMC160229

[17] Kuluev BR, Safiullina MG, Knyazev AV, et al Effect of ectopic expression of *NtEXPA5* gene on cell size and growth of organs of transgenic tobacco plants. Russ J Dev Biol. 2013;44:28–34. 10.7868/s0475145013010059 23659080

[18] Cosgrove DJ Enzymes and other agents that enhance cell wall extensibility. Annu Rev Plant Physiol Plant Mol Biol. 1999;50:391–417. 11541953 10.1146/annurev.arplant.50.1.391

[19] Cosgrove DJ, Durachko DM Autolysis and extension of isolated walls from growing cucumber hypocotyls. J Exp Bot. 1994;45:1711–1719. 11540379 10.1093/jxb/45.special_issue.1711

[20] McQueen-Mason SJ, Cosgrove DJ Expansin mode of action on cell walls. Analysis of wall hydrolysis, stress relaxation, and binding. Plant Physiol. 1995;107:87–100. 11536663 10.1104/pp.107.1.87 PMC161171

[21] Kende H, Bradford K, Brummell D, et al Nomenclature for members of the expansin superfamily of genes and proteins. Plant Mol Biol. 2004;55:311–314. 15604683 10.1007/s11103-004-0158-6

[22] Bunterngsook B, Mhuantong W, Champreda V, et al Identification of novel bacterial expansins and their synergistic actions on cellulose degradation. Bioresour Technol. 2014;159:64–71. 24632627 10.1016/j.biortech.2014.02.004

[23] Wolf S, Hématy K, Höfte H Growth control and cell wall signaling in plants. Annu Rev Plant Biol. 2012;63:381–407. 22224451 10.1146/annurev-arplant-042811-105449

[24] Cosgrove DJ Plant cell enlargement and the action of expansins. BioEssays News Rev Mol Cell Dev Biol. 1996;18:533–540. 10.1002/bies.950180704 8757932

[25] Jahr H, Dreier J, Meletzus D, et al The endo-beta-1,4-glucanase CelA of Clavibacter michiganensis subsp. michiganensis is a pathogenicity determinant required for induction of bacterial wilt of tomato. Mol Plant Microbe Interact. 2000;13:703–714. 10875331 10.1094/MPMI.2000.13.7.703

[26] Li Y, Darley CP, Ongaro V, et al Plant expansins are a complex multigene family with an ancient evolutionary origin. Plant Physiol. 2002;128:854–864. 11891242 10.1104/pp.010658 PMC152199

[27] Saloheimo M, Paloheimo M, Hakola S, et al Swollenin, a *Trichoderma reesei* protein with sequence similarity to the plant expansins, exhibits disruption activity on cellulosic materials. Eur J Biochem. 2002;269:4202–4211. 12199698 10.1046/j.1432-1033.2002.03095.x

[28] Shcherban TY, Shi J, Durachko DM, et al Molecular cloning and sequence analysis of expansins–a highly conserved, multigene family of proteins that mediate cell wall extension in plants. Proc Natl Acad Sci U S A. 1995;92:9245–9249. 7568110 10.1073/pnas.92.20.9245 PMC40961

[29] Yennawar NH, Li L-C, Dudzinski DM, et al Crystal structure and activities of EXPB1 (Zea m 1), a β-expansin and group-1 pollen allergen from maize. Proc Natl Acad Sci U S A. 2006;103:14664–14671. 16984999 10.1073/pnas.0605979103 PMC1595409

[30] Nikolaidis N, Doran N, Cosgrove DJ Plant expansins in bacteria and fungi: evolution by horizontal gene transfer and independent domain fusion. Mol Biol Evol. 2014;31:376–386. 24150040 10.1093/molbev/mst206

[31] Georgelis N, Nikolaidis N, Cosgrove DJ Bacterial expansins and related proteins from the world of microbes. Appl Microbiol Biotechnol. 2015;99:3807–3823. 25833181 10.1007/s00253-015-6534-0 PMC4427351

[32] Zdobnov EM, Apweiler R InterProScan–an integration platform for the signature-recognition methods in InterPro. Bioinformatics. 2001;17:847–848. 11590104 10.1093/bioinformatics/17.9.847

[33] Mi H, Dong Q, Muruganujan A, et al PANTHER version 7: improved phylogenetic trees, orthologs and collaboration with the gene ontology consortium. Nucleic Acids Res. 2009;38:D204–10. 20015972 10.1093/nar/gkp1019 PMC2808919

[34] Cosgrove DJ Plant expansins: diversity and interactions with plant cell walls. Curr Opin Plant Biol. 2015;25:162–172. 26057089 10.1016/j.pbi.2015.05.014 PMC4532548

[35] Georgelis N, Yennawar NH, Cosgrove DJ Structural basis for entropy-driven cellulose binding by a type-A cellulose-binding module (CBM) and bacterial expansin. Proc Natl Acad Sci U S A. 2012;109:14830–14835. 22927418 10.1073/pnas.1213200109 PMC3443152

[36] Georgelis N, Tabuchi A, Nikolaidis N, et al Structure-function analysis of the bacterial expansin EXLX1. J Biol Chem. 2011;286:16814–16823. 21454649 10.1074/jbc.M111.225037 PMC3089525

[37] Armijos-Jaramillo V, Santander-Gordón D, Soria R, et al A whole genome analysis reveals the presence of a plant PR1 sequence in the potato pathogen *Streptomyces scabies* and other *Streptomyces* species. Mol Phylogenet Evol. 2016;114:346–352. 27530704 10.1016/j.ympev.2016.08.006

[38] Vernikos GS, Parkhill J Interpolated variable order motifs for identification of horizontally acquired DNA: revisiting the Salmonella pathogenicity islands. Bioinforma Oxf Engl. 2006;22:2196–2203. 10.1093/bioinformatics/btl369 16837528

[39] Bertelli C, Laird MR, Williams KP Simon fraser university research computing group, Lau BY, Hoad G, Winsor GL, Brinkman FSL. IslandViewer 4: expanded prediction of genomic islands for larger-scale datasets. Nucleic Acids Res. 2017;45:W30–W5. 28472413 10.1093/nar/gkx343 PMC5570257

[40] Katoh K, Standley DM MAFFT multiple sequence alignment software version 7: improvements in performance and usability. Mol Biol Evol. 2013;30:772–780. 23329690 10.1093/molbev/mst010 PMC3603318

[41] Guindon S, Dufayard J-F, Lefort V, et al New algorithms and methods to estimate maximum-likelihood phylogenies: assessing the performance of PhyML. 3.0. Syst Biol. 2010;59:307–321. 20525638 10.1093/sysbio/syq010

[42] Capella-Gutiérrez S, Silla-Martínez JM, Gabaldón T trimAl: a tool for automated alignment trimming in large-scale phylogenetic analyses. Bioinformatics. 2009;25:1972–1973. 19505945 10.1093/bioinformatics/btp348 PMC2712344

[43] Talavera G, Castresana J Improvement of phylogenies after removing divergent and ambiguously aligned blocks from protein sequence alignments. Syst Biol. 2007;56:564–577. 17654362 10.1080/10635150701472164

[44] Larkin MA, Blackshields G, Brown NP, et al Clustal W and Clustal X version. 2.0. Bioinform. 2007;23:2947–2948. 10.1093/bioinformatics/btm404 17846036

[45] Edgar RC MUSCLE: multiple sequence alignment with high accuracy and high throughput. Nucleic Acids Res. 2004;32:1792–1797. 15034147 10.1093/nar/gkh340 PMC390337

[46] Wattam AR, Davis JJ, Assaf R, et al Improvements to PATRIC, the all-bacterial bioinformatics database and analysis resource center. Nucleic Acids Res. 2017;45:D535–42. 27899627 10.1093/nar/gkw1017 PMC5210524

[47] Kumar S, Stecher G, Suleski M, et al TimeTree: a resource for timelines, timetrees, and divergence times. Mol Biol Evol. 2017;34:1812–1819. 28387841 10.1093/molbev/msx116

[48] Buchan DWA, Minneci F, Nugent TCO, et al Scalable web services for the PSIPRED protein analysis workbench. Nucleic Acids Res. 2013;41:W349–W357. 23748958 10.1093/nar/gkt381 PMC3692098

[49] Jones DT Protein secondary structure prediction based on position-specific scoring matrices. J Mol Biol. 1999;292:195–202. 10493868 10.1006/jmbi.1999.3091

[50] Lobley A, Sadowski MI, Jones DT pGenTHREADER and pDomTHREADER: new methods for improved protein fold recognition and superfamily discrimination. Bioinformatics. 2009;25:1761–1767. 19429599 10.1093/bioinformatics/btp302

[51] Pettersen EF, Goddard TD, Huang CC, et al UCSF Chimera–a visualization system for exploratory research and analysis. J Comput Chem. 2004;25:1605–1612. 15264254 10.1002/jcc.20084

[52] Webb B, Sali A Comparative protein structure modeling using MODELLER. Curr Protoc Bioinforma. 2016;54:5.6.1–5.6.37. 10.1002/cpbi.3 PMC5031415 27322406

[53] Jurrus E, Engel D, Star K, et al Improvements to the APBS biomolecular solvation software suite. Protein Sci. 2018;27:112–128. 28836357 10.1002/pro.3280 PMC5734301

[54] Berman HM, Westbrook J, Feng Z, et al The protein data bank. Nucleic Acids Res. 2000;28:235–242. 10592235 10.1093/nar/28.1.235 PMC102472

